# Prevalence and Prognostic Value of Heart Failure Stages: An Elderly Inpatient Based Cohort Study

**DOI:** 10.3389/fmed.2021.639453

**Published:** 2021-04-22

**Authors:** Pei-Pei Zheng, Si-Min Yao, Di Guo, Ling-ling Cui, Guo-Bin Miao, Wei Dong, Hua Wang, Jie-Fu Yang

**Affiliations:** ^1^Department of Cardiology, Beijing Hospital, National Center of Gerontology, Institute of Geriatric Medicine, Chinese Academy of Medical Sciences, Beijing, China; ^2^Department of Cardiology, Tsinghua University Affiliated Beijing Tsinghua Changgung Hospital, Beijing, China; ^3^Department of Cardiology, Chinese PLA General Hospital, Medical School of Chinese PLA, Beijing, China

**Keywords:** heart failure stages, epidemiology, prognosis, NT-proBNP, elderly inpatients

## Abstract

**Background:** The prevalence and prognostic value of heart failure (HF) stages among elderly hospitalized patients is unclear.

**Methods:** We conducted a prospective, observational, multi-center, cohort study, including hospitalized patients with the sample size of 1,068; patients were age 65 years or more, able to cooperate with the assessment and to complete the echocardiogram. Two cardiologists classified all participants in various HF stages according to 2013 ACC/AHA HF staging guidelines. The outcome was rate of 1-year major adverse cardiovascular events (MACE). The Kaplan–Meier method and Cox proportional hazards models were used for survival analyses. Survival classification and regression tree analysis were used to determine the optimal cutoff of N-terminal pro-brain natriuretic peptide (NT-proBNP) to predict MACE.

**Results:** Participants' mean age was 75.3 ± 6.88 years. Of them, 4.7% were healthy and without HF risk factors, 21.0% were stage A, 58.7% were stage B, and 15.6% were stage C/D. HF stages were associated with worsening 1-year survival without MACE (log-rank χ^2^ = 69.62, *P* < 0.001). Deterioration from stage B to C/D was related to significant increases in HR (3.636, 95% CI, 2.174–6.098, *P* < 0.001). Patients with NT-proBNP levels over 280.45 pg/mL in stage B (HR 2; 95% CI 1.112–3.597; *P* = 0.021) and 11,111.5 pg/ml in stage C/D (HR 2.603, 95% CI 1.014–6.682; *P* = 0.047) experienced a high incidence of MACE adjusted for age, sex, and glomerular filtration rate.

**Conclusions :** HF stage B, rather than stage A, was most common in elderly inpatients. NT-proBNP may help predict MACE in stage B.

**Trial Registration:** ChiCTR1800017204; 07/18/2018.

## Introduction

Heart failure (HF) stages are progressive. Once HF progresses, patients seldom return to lower stages (1). Despite early recognition and prevention of HF, the increasing prevalence of risk factors (hypertension, diabetes, and obesity) along with the aging of the population and the prevalence of unhealthy lifestyles have increased the risk of HF (2–4). HF stages A and B were found to be more common in the elderly (83.4%) than in young adults (58.7%) in the community (5). The risk of HF was higher in the hospital than in the community; nevertheless, there are few data on the prevalence of HF stages among older hospitalized adults. Cardiac parameters measured by echocardiogram change with age; these changes include smaller left ventricular (LV) size, higher LV ejection fraction (LVEF), and lower early diastolic mitral annular velocity (e′) (6, 7); nevertheless, there are few data on elderly Asian populations with HF confirmed by echocardiography.

Reduced survival and increased brain natriuretic peptide levels are closely related to the progression of HF (5). Five-year mortality after hospitalization for decompensated HF is more than 75% (8). As individuals are living longer, it is essential to evaluate the risk of HF, including cardiovascular-related events. Outcomes of clinical HF have been adequately explored (9–11); nevertheless, there are few data regarding outcomes in elderly inpatients at various HF stages.

The prevalence and prognostic value of HF stages among elderly inpatients is unclear. Therefore, the objectives of our study were as follows: (1) to measure the prevalence of HF stages in an elderly inpatient-based cohort; (2) to measure the predictive value of HF stages; and (3) to determine the level of N-terminal pro-brain natriuretic peptide (NT-proBNP) that would predict outcome in various HF stages.

## Methods

### Study Setting and Study Population

**Figure 1:**
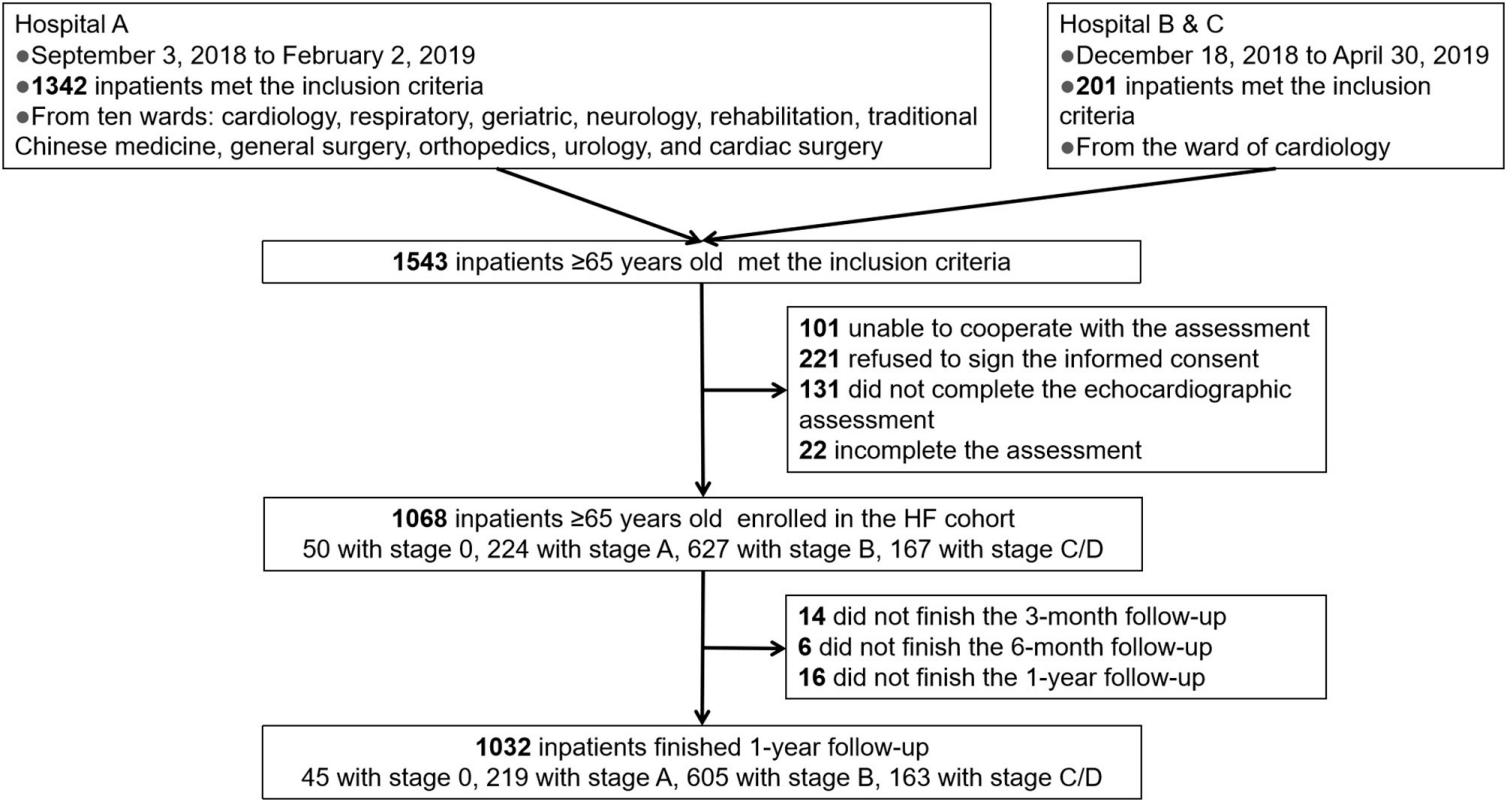
Flow chart of the HF cohort. HF, heart failure.

This trial was conducted in accordance with the Declaration of Helsinki, and was approved by the Ethics Committee of Beijing Hospital (approval no. 2018BJYYEC-121-02). Our previous elderly inpatients' cohort study on frailty screened 1,543 elderly inpatients who were consecutively admitted to ten wards covering medical and surgical departments (cardiology, respiratory, geriatric, neurology, rehabilitation, traditional Chinese medicine, general surgery, orthopedics, urology, and cardiac surgery) in three tertiary referral hospitals in Beijing, China, from September 2018 to April 2019. We excluded the inpatients failed to complete the echocardiogram based on the previous inclusion criterion (aged 65 years or older; hospitalized patients) and Exclusion criteria (inability to cooperate with the assessment; refusal to sign the informed consent form). Finally, a total of 1,068 patients provided written consent and enrolled in the HF cohort to be followed up for 1 year, while 475 met the exclusion criteria (Figure 1). Age, sex, years of education, risk factors for HF, myocardial infarction, blood pressure, NT-proBNP level, hemoglobin A1c (HbA1c) level, internal medicine operations and surgery operations were obtained from the medical records. All related diseases were based on the diagnosis of physicians and the treatment records. Hypertension control was defined when systolic blood pressure was 130–140 mmHg for those aged 65 years or older, 120–130 mmHg for those with LV hypertrophy, and <140 mmHg for those with stage C/D HF, according to 2018 European Society of Cardiology/European Society of Hypertension guidelines for the management of arterial hypertension (12). HbA1c control was defined as HbA1c 6.5–7% in those without severe diabetes complications, and 7–8% for those with severe diabetes complications, according to the 2019 American Heart Association/Heart Failure Society of American type 2 diabetes mellitus and heart failure guidelines (13). Estimated glomerular filtration rate (EGFR) was calculated using the 2009 CKD-EPI creatinine equation (14). Data were managed through Research Electronic Data Capture and the entire study was supervised by Peking University Clinical Research Institute.

### Definition of HF Stages

HF stages were determined by two cardiologists according to the 2013 American College of Cardiology/American Heart Association (ACC/AHA) Guidelines for the Evaluation and Management of Chronic Heart Failure in the Adult (1).

Stage 0 means healthy and without HF risk factors.

Stage A means high risk for HF but without structural and functional heart disease or symptoms of HF. Risk factors include hypertension, diabetes mellitus, obesity (Body mass index ≥30 kg/m^2^), and coronary artery disease (without myocardial infarction).

Stage B means structural and functional heart disease that is strongly associated with the development of HF but without HF signs or symptoms, including valvular heart disease, myocardial infarction (wall-motion abnormalities), LV hypertrophy, LV enlargement, systolic dysfunction, and diastolic dysfunction (5). Valvular heart disease was defined as moderate or greater stenosis or regurgitation in the aortic or mitral valve, wall-motion abnormalities were defined as hypokinesis, akinesis, or dyskinesis of two or more contiguous segments of the LV. LV hypertrophy was defined as LV mass/height^2.7^ > 45 g/m^2.7^ (men) or > 41.5 g/m^2.7^ (women), and LV enlargement was defined as LV end-diastolic volume /body surface area > 60.2 mL/m^2^ (men) or > 51.9 mL/m^2^ (women) (15). Systolic dysfunction was defined as LVEF, measured by biplane Simpsons <54.6% (men aged 65–69 years), <53% (men aged ≥70 years), <54.5% (women aged 65–69 years), or <53.5% (women aged ≥ 70 years). Diastolic dysfunction was defined as having one of the following: (1) septal e′ <4.3 cm/s (men) or <4.1 cm/s (women); (2) septal E/e′ ratio > 14.8 (men) or > 17.4 (women); 3) left atrial anteroposterior diameter > 39.2 mm (men aged 65–69 years), > 40.3 mm (men aged ≥ 70 years), > 38.3 mm (women aged 65–69 years), or > 38.6 mm (women aged ≥ 70 years) (16).

Stage C/D means structural heart disease with prior or current symptoms of HF whether requiring specialized interventions or not.

### Endpoint

The composite endpoint was 1-year major adverse cardiovascular events (MACE), defined as the classical compound endpoint of non-fatal myocardial infarction, non-fatal stroke or cardiovascular death (17), and other cardiovascular events requiring hospitalization (unstable angina, severe arrhythmia, HF) (18). We confirmed the MACE occurrence through telephone interviews with the participant or caregiver 1 year after the date of signing informed consent, and through medical record review, if necessary.

### Statistical Analysis

Shapiro–Wilk tests and quantile–quantile plots were used to evaluate continuous variables for normal distribution. Categorical variables were expressed as percentages, and comparison between groups were made using Pearson χ^2^ tests. Normally distributed continuous variables were expressed as mean ± standard deviation. Comparisons between groups were performed using ANOVA and *post hoc* tests were employed to detect significant pairs. Non-normally distributed continuous variables were expressed as median (interquartile range: 25th to 75th percentiles), and the Mann–Whitney *U*-test was used to compare groups.

Survival without MACE was estimated using the Kaplan–Meier method. Comparisons between groups were based on the log-rank test for univariate analyses and Cox proportional hazards models when adjusting for confounders. Confounders were age and sex for the Cox model of MACE and HF stages. Age, sex, and EGFR were confounders for the Cox model of MACE and NT-proBNP at various HF stages. The results of survival analyses were presented as hazard ratios (HR) and 95% confidence intervals (CIs). The best NT-proBNP cut-off point predicting MACE was determined by survival classification and regression tree (CART) analysis.

Two-tailed *P*-value <0.05 was considered statistically significant. Data were analyzed using SPSS software version 25 (IBM Corp., Armonk, NY, USA) and figures were generated using Graph Pad Prism version 6.01 (Graph Pad Software Inc., San Diego, CA, USA).

## Results

### Prevalence of HF Stages

**Figure 2:**
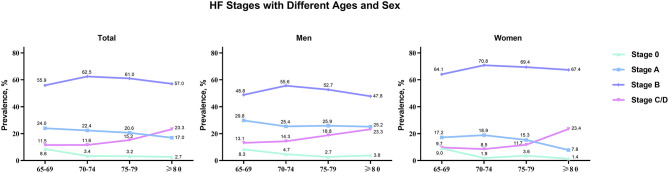
HF stages with different ages (65–69, 70–74, 75–79, and ≥80) and sex (total, men, and women).

There were 1,068 elderly inpatients enrolled in the cohort; 50 (4.7%) inpatients were classified as normal (stage 0), 224 (21.0%) were stage A, 627 (58.7%) were stage B, and 167 (15.6%) were stage C/D (Figure 2). The prevalence increased with advancing age for stage C/D, for stage B before 75 years, and decreased with advancing age for stages 0 and A. The age distribution of HF stages was similar in men and women.

### Characteristics of HF Stages

Characteristics of HF stages are displayed in Table 1. Mean age 75.3 ± 6.88 years, 47% were women and age increased with HF stage. Years of education decreased with increasing HF stage. The current smoking rate was low. The majority had preserved LVEF (92.0%) and hypertension (72.8%), while only 29.5% with hypertension had it well-controlled.

**Table 1:** Baseline and heart failure characteristics of all participants.

	**Overall**	**Stage 0**	**Stage A**	**Stage B**	**Stage C/D**	***P*-values**
	***n* = 1,068**	***n* = 50 (4.7%)**	***n* = 224 (21.0%)**	***n* = 627 (58.7%)**	***n* = 167 (15.6%)**	
Age, years	75.3 ± 6.88	72.3 ± 6.99	74.3 ± 6.64	75.3 ± 6.62 ^*^	77.6 ± 7.50 ^*^^†^^‡^	<0.001
Women	503 (47.1)	21 (42.0)	73 (32.6)	340 (54.2)^†^	69 (41.3)^‡^	<0.001
Education, years	10.9 ± 4.46	11.9 ± 4.02	11.7 ± 4.01	10.8 ± 4.42	9.85 ± 5.05 ^*^^‡^	<0.001
Current smoker	98 (9.2)	6 (12)	27 (12.1)	53 (8.5)	12 (7.2)	0.273
**Risk factors for HF**						
Hypertension	777 (72.8)	0 (0)	156 (69.6)^*^	492 (78.5)^*^^†^	129 (77.2)^*^	<0.001
Hypertension control	229 (29.5)	–	44 (28.2)	92 (18.7)^†^	93 (72.1)^†^^‡^	<0.001
Diabetes	369 (34.6)	0 (0)	84 (37.5)^*^	211 (33.7)^*^	74 (44.3)^*^	<0.001
HbA1c control	100 (27.1)	–	25 (29.8)	56 (26.5)	19 (25.7)	0.835
Obesity	203 (19.0)	0 (0)	36 (16.1)^*^	140 (22.3)^*^	27 (16.2)^*^	<0.001
Coronary heart disease	609 (57.0)	0 (0)	125 (55.8)^*^	367 (58.5)^*^	117 (70.1)^*^^†^^‡^	<0.001
**Reasons for stage B HF**						
Valvular heart disease	76 (7.1)	–	–	38 (6.1)^†^	38 (22.8)^*^^†^^‡^	<0.001
Myocardial infarction (wall-motion abnormalities)	162 (15.2)	–	–	97 (15.5)^*^^†^	65 (38.9)^*^^†^^‡^	<0.001
LV hypertrophy	432 (40.4)	–	–	329 (52.5)^*^^†^	103 (61.7)^*^^†^^‡^	<0.001
LV enlargement	425 (39.7)	–	–	340 (54.2)^*^^†^	85 (50.9)^*^^†^	<0.001
Systolic dysfunction	103 (9.6)	–	–	30 (4.8)^†^	73 (43.7)^*^^†^^‡^	<0.001
Diastolic dysfunction	422 (39.5)	–	–	316 (50.4)^*^^†^	106 (63.5)^*^^†^^‡^	<0.001
**Other concerns**						
LVEF ≥50	983 (92.0)	50 (100)	224 (100)	614 (97.9)	95 (56.8) ^*^^†^^‡^	<0.001
EGFR <60 ml/(min × 1.73^2^)	199 (18.6)	4 (8.0)	17 (7.6)	100 (15.9)^†^	78 (46.7)^*^^†^^‡^	<0.001
NT-proBNP, pg/ml	173 [80.4, 582]	86.8 [59.8, 169]	94.6 [55.1, 176]	164 [80, 388]^†^	1,474 [520, 2,326]^*^^†^^‡^	<0.001
Internal medicine operations	445 (41.7)	7 (14.0)	94 (42.0)^*^	283 (45.1)^*^	61 (36.5)^*^	<0.001
Surgery operations	172 (16.1)	19 (38.0)	42 (18.8)^*^	100 (15.9)^*^	11 (6.6)^*^^†^^‡^	<0.001

*Values are shown as mean ± standard deviation or median [interquartile range: 25th to 75th percentiles] or n (%)*.

*HF, heart failure; HbA1c, hemoglobin A1c; LV, left ventricular; LVEF, left ventricular eject fraction; EGFR, estimated glomerular filtration rate; NT-proBNP, N-terminal pro-B-type natriuretic peptide*.

* *P <0.05 compared with Stage 0*;

† *P <0.05 compared with Stage A*;

‡ *P <0.05 compared with Stage B*.

For patients in stage A, we examined the range assignment of HF risk factors: 69.6% had hypertension, 37.5% had diabetes, 16.1% were obese, and 55.8% had coronary heart disease. All patients had preserved LVEF. The inpatients with stage A received less internal medicine operations (χ^2^ = 20.67, *P* < 0.001) and more surgery operations (χ^2^ = 30.11 *P* < 0.001) than HF stages with B/C/D.

For patients in stage B, the distribution of structural or functional abnormalities was as follows: 6.1% with valvular heart disease; 15.5% with myocardial infraction and wall-motion abnormalities; 52.5% with LV hypertrophy; 54.2% with LV enlargement; 4.8% with systolic dysfunction; and 50.4% with diastolic dysfunction. Patients with hypertension had significantly worse blood pressure control than did patients in stage A (χ^2^ = 6.45, *P* = 0.011) or stage C/D (χ^2^ = 139.31, *P* < 0.001).

For patients in stage C/D, coronary heart disease (χ^2^ = 7.36, *P* = 0.007), LV hypertrophy (χ^2^ = 11.09, *P* = 0.004), systolic dysfunction (χ^2^ = 186.76, *P* < 0.001), and diastolic dysfunction (χ^2^ = 43.74, *P* < 0.001) accounted for more than a half and were significantly higher than in patients in stage B. The proportion of patients with EGFR <60 ml/ (min × 1.73 m^2^) was significantly greater in stage C/D than in other HF stages (stage C/D 46.7% vs. stage 0 8% vs. stage A 7.6% vs. stage B 15.9%, *P* < 0.001).

### HF Stages and 1-year MACE

**Figure 3:**
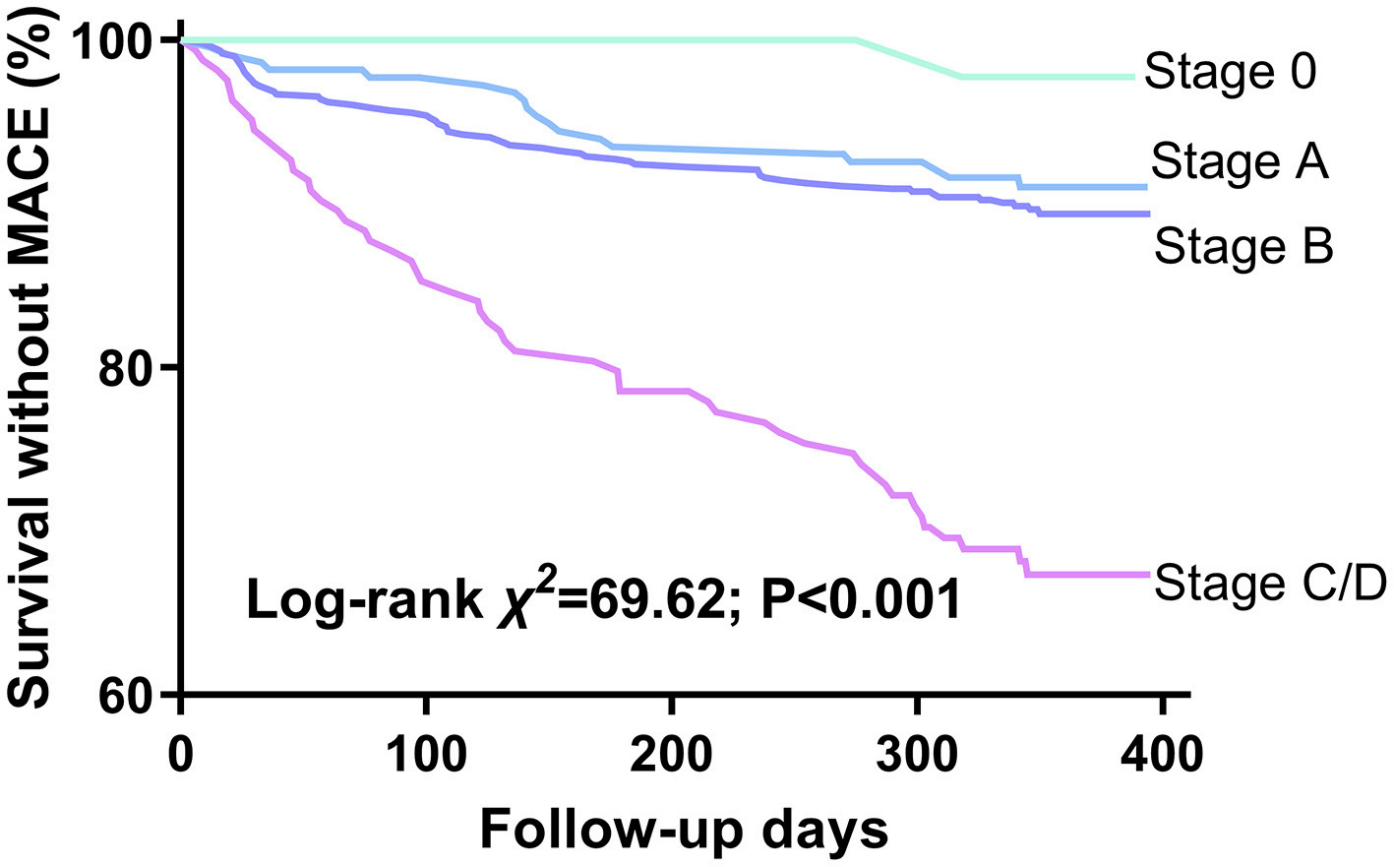
Kaplan-Meier survival curves by HF stages. Events are defined by 1-year MACE. MACE, major adverse cardiovascular events.

During the follow-up period, MACE occurred in 138 (12.9%) patients. HF stages were associated with progressively worsening 1-year survival without MACE (Figure 3, log-rank χ^2^ = 69.62, *P* < 0.001) and increasing 1-year MACE rate: stage 0, 2%; stage A, 9.4%; stage B, 10%; and stage C/D, 31.7% (χ^2^ = 64.95, *P* < 0.001). HF stages A through C/D were associated with progressively increasing MACE HRs compared with stage 0 when adjusted for age and sex: stage A, 4.501 (95% CI, 0.605–33.477, *P* = 0.142); stage B, 5.008 (95% CI, 0.693–36.165, *P* = 0.110); and stage C/D, 16.427 (95% CI, 2.261–119.332, *P* = 0.006). Only deterioration from stage B to C/D was associated with significant incremental increases in HR: stage C/D → B, HR = 3.636 (95% CI, 2.174–6.098, *P* < 0.001), while stage B → A, HR = 1.116 (95% CI, 0.678–1.838, *P* = 0.667).

### NT-proBNP Levels and 1-Year MACE

**Figure 4:**
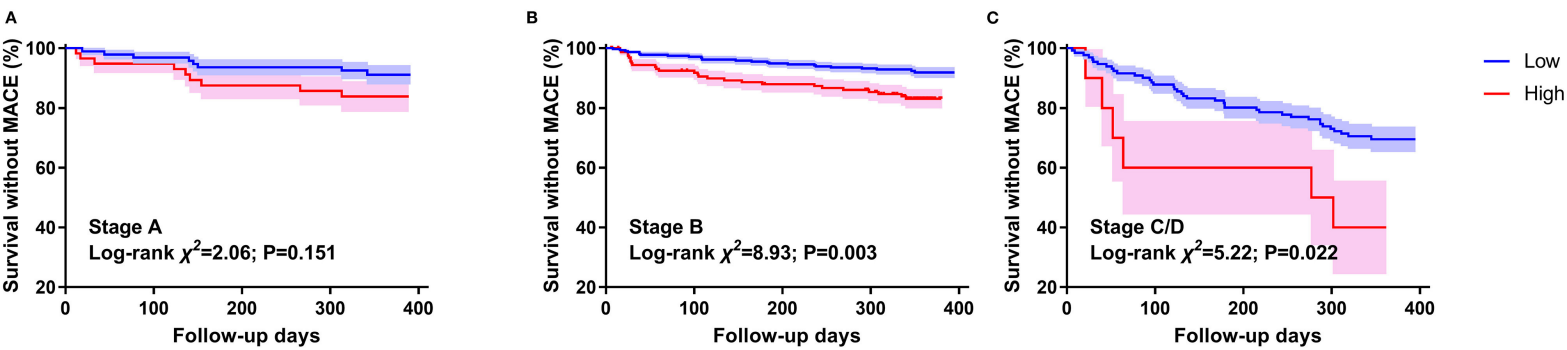
Kaplan–Meier survival curves by NT-proBNP in different HF stages. **(A)** stands for patients with stage A HF. **(B)** stands for patients with stage B HF. **(C)** stands for patients with stage C/D HF. Events are defined by 1-year MACE. The NT-proBNP cut-off values between high and low are 121.9 pg/ml for stage A, 280.45 pg/ml for stage B, and 11,111.5 pg/ml for stage C/D.

The survival CART analysis revealed that the NT-proBNP cut-off points for predicting MACE were as follows: 121.9 pg/ml for stage A, 280.45 pg/ml for stage B, and 11,111.5 pg/ml for stage C/D. We found that patients with NT-proBNP level over 280.45 pg/mL in stage B and 11,111.5 pg/ml in stage C/D experienced a high incidence of MACE with (Figure 4B, log-rank χ^2^ = 8.928, *P* < 0.003; Figure 4C, log-rank χ^2^ = 5.224, *P* = 0.022) and without (stage B, HR 2; 95% CI 1.112–3.597; *P* = 0.021; stage C/D, HR 2.603, 95% CI 1.014–6.682; *P* = 0.047) adjusting for age, sex, and EGFR, rather than NT-proBNP level over 121.9 pg/mL in stage A (Figure 4A, *P* > 0.05).

## Discussion

We applied the 2013 ACC/AHA HF staging in Asian inpatients aged 65 years or older to determine the risk factors and functional and structural ventricular dysfunctions associated with HF. In our cohort, 4.7% inpatients were classified as normal (stage 0), 21.0% demonstrated HF risk factors (stage A), 58.7% had asymptomatic functional or structural abnormalities (stage B), and 15.6% presented with overt HF symptoms (stage C/D). HF stages were associated with progressively higher 1-year risk of MACE.

### HF Stages

Chamber remodeling, symptomatic HF, and high disability and Mortality mutually worsen one another (19–21). Early diagnosis and intervention are recommended to prevent HF development (22, 23).

With the increase of age, the prevalence of HF risk factors, the distribution of HF stages in elderly is different from that in young adults. Among 6,118 participants in the Atherosclerosis Risk in Communities study (age 67–91 years) from United States, 52% were categorized as stage A and 30% were stage B (24). Similarly, in the Characteristics and Course of HF Stages A–B and Determinants of Progression cohort study (915 participants aged 60 years or more in community), 55.5% were stage A and 23.6% were stage B (25). Compared with community-based patients, we found that inpatients had markedly higher prevalence of Stage B HF (58.7%) and lower prevalence of Stage A (21%). The prevalence increased with advancing age for stage B before 75 years of age, and decreased with advancing age for stages 0 and A. These age- and location-related changes between stages A and B may account for the increase in the incidence and prevalence of clinical HF in the hospital, especially in perioperative period.

### Outcomes

Asymptomatic precursor stages can help predict development of clinical HF and survival (5, 24, 26). This is the basis of the primary prevention of HF. We found that the risk of MACE increased with HF stages and that the risk significantly increased from stage B to C/D among elderly inpatients, suggesting that the recognition and treatment of stage B is critical.

The Copenhagen HF risk study found that 37.5% of stage A inpatients were misdiagnosed, and they were being in stage B actually (27). It is important recognize stage B HF in a timely fashion. The combination of use of the Atherosclerosis Risk in Communities criteria and the Echocardiographic Measurements in Normal Chinese Adults criteria is useful to recognize stage B HF among elderly inpatients, as shown in our study.

Hypertension, coronary heart disease, and preserved LVEF were the main features of stage B HF in our cohort. Previous studies suggested that the increasing prevalence of hypertension may account for this (28). However, we found that individuals with hypertension and stage B had significantly worse blood pressure control, suggesting that adequate blood pressure control (not too low or too high) may prevent the progression of HF. Another study found that most evidence-based cardiac prevention therapies were used in fewer than 60% of patients (29), and that these may also useful for prevention. We found an increasing proportion of EGFR from stage B to stage C/D. Chronic kidney disease has been shown to be a risk factor for clinical HF. For this reason, protection of renal function should not be ignored.

NT-proBNP and BNP levels have been shown to be associated with subsequent HF and death in patients with cardiovascular heart disease or HF (30, 31). Use of these parameters has been advocated for screening and supporting clinical decision making for patients with HF (1, 32). We found that NT-proBNP increased with HF stage and that it predicted 1-year MACE in elderly inpatients with stages B and C/D. To recognize individuals at high risk for MACE in stages B and C/D, we suggest using the cutoff points of 280.45 pg/ml and 11,111.5 pg/ml, respectively. The STOP-HF randomized trial found that patients at high risk of HF can avoid clinical HF after intervention when their BNP levels were more than 50 ng/ml; however, they focused on BNP and their participants were older than 45 years (33). Notably, our findings suggest a NT-proBNP level, which was more stable, in patients with stage B HF and aged 65 years or more.

### Limitations

The current study did not have a sufficiently long follow-up. Of note, our participants were all form the ordinary ward rather than the emergency ward or intensive care unit. Given that patients with acute HF usually were hospitalized in emergency ward or intensive care unit, the Therefore, the sample of patients with stage C/D HF were small that limits our making meaningful observations about clinical HF. Nearly 30% of the elderly inpatients, who fulfill the inclusion criterion, not agreed to participant our present study increases the recruitment bias. The focus on elderly inpatients may limit the generalization to younger adults and those in the community. Renal function plays an important role in the development of HF, however, the focus on the EGFR ignored other evaluation of renal function, such as albuminuria. Studies with larger sample sizes, longer follow-up, less recruitment bias, information on drug therapy and renal functional evaluation, and specific heart disease-related endpoints are warranted in the future. Given that the prevalence of HF stages is highly dependent on reasons of admission to the hospital, further studies are also needed to be conducted to clarify the heart failure stages in a specific department or disease to guide accurate strategies.

## Conclusion

Among inpatients aged 65 years or more, the high prevalence of stage B HF, with the characters of LV hypertrophy, LV enlargement; diastolic dysfunction, hypertension and worse blood pressure control, and worsening outcomes from stage B to C/D need to be areas of focus. NT-proBNP is useful to identify individuals at high risk for MACE in stage B HF.

## Data Availability Statement

The original contributions presented in the study are included in the article/supplementary material, further inquiries can be directed to the corresponding authors.

## Ethics Statement

The studies involving human participants were reviewed and approved by Ethics Committee of Beijing Hospital. The patients/participants provided their written informed consent to participate in this study.

## Author Contributions

P-PZ and S-MY collected data, analyzed the data, and wrote the manuscript. DG and L-lC collected data and wrote the manuscript. G-BM and WD collected data and revised the manuscript. HW and J-FY provided the study plan (concept and design) and revised the manuscript. All authors contributed to the article and approved the submitted version.

## Conflict of Interest

The authors declare that the research was conducted in the absence of any commercial or financial relationships that could be construed as a potential conflict of interest.
